# Leptospirosis in Indonesia: diagnostic challenges associated with atypical clinical manifestations and limited laboratory capacity

**DOI:** 10.1186/s12879-020-4903-5

**Published:** 2020-02-27

**Authors:** Muhammad Hussein Gasem, Usman Hadi, Bachti Alisjahbana, Emiliana Tjitra, M. M. D. E. A. H. Hapsari, Endang Sri Lestari, Abu Tholib Aman, Dewi Lokida, Gustiani Salim, Herman Kosasih, Ketut Tuti Parwati Merati, Kanti Laras, Mansyur Arif, Nurhayati Lukman, Pratiwi Sudarmono, Vivi Lisdawati, Chuen-Yen Lau, Aaron Neal, Muhammad Karyana

**Affiliations:** 1 Dr. Kariadi Hospital – Faculty of Medicine, Diponegoro University, Semarang, Indonesia; 2 Dr. Soetomo Academic General Hospital, Faculty of Medicine Airlangga University, Surabaya, Indonesia; 3 Hasan Sadikin Hospital – Faculty of Medicine, Padjadjaran University, Bandung, Indonesia; 4 National Institute of Health Research and Development (NIHRD), Ministry of Health Republic of Indonesia, Jakarta, Indonesia; 5 Dr. Sardjito Hospital – Faculty of Medicine, Public Health and Nursing, Gadjah Mada University, Yogyakarta, Indonesia; 6 Tangerang District Hospital, Tangerang, Indonesia; 7 Indonesia Research Partnership on Infectious Disease (INA-RESPOND), Badan Litbangkes, Building 4, 5th floor, Jl Percetakan Negara no 29, Jakarta, 10560 Indonesia; 8 Department of Internal Medicine, Udayana University, Bali, Indonesia; 9 Dr. Wahidin Sudirohusodo Hospital, Makassar, Indonesia; 10 Dr. Cipto Mangunkusumo Hospital, Jakarta, Indonesia; 11 Prof. Dr. Sulianti Saroso Hospital, Jakarta, Indonesia; 12 National Institute of Allergy and Infectious Diseases (NIAID), National Institutes of Health, Bethesda, MD USA

**Keywords:** Leptospirosis, Diagnostic challenge, Atypical manifestations, Indonesia

## Abstract

**Background:**

The burden of leptospirosis in Indonesia is poorly understood. Data from an observational study conducted from 2013 to 2016 in seven cities across Indonesia was used to estimate the incidence of leptospirosis and document its clinical manifestations in patients requiring hospitalization.

**Methods:**

Specimens from patients hospitalized with acute fever were collected at enrollment, 14–28 days, and 3 months. Demographic and clinical information were collected during study visits and/or retrieved from medical records and double-entered into clinical report forms. After initially screening for dengue virus and other pathogens, specimens were tested at a central Reference Laboratory for anti-*Leptospira* IgM using commercial ELISA kits and for *Leptospira* DNA using an in-house quantitative real-time PCR assay.

**Results:**

Of 1464 patients enrolled, 45 (3.1%) confirmed cases (by PCR and/or sero-coversion or four-fold increase of IgM) and 6 (0.4%) probable cases (by high titer IgM) of leptospirosis were identified by the Reference Laboratory. Disease incidence at sites ranged from 0 (0%) cases in Denpasar to 17 (8.9%) cases in Semarang. The median age of patients was 41.2 years (range of 5.3 to 85.0 years), and 67% of patients were male. Twenty-two patients (43.1%) were accurately diagnosed at sites, and 29 patients (56.9%) were clinically misdiagnosed as having another infection, most commonly dengue fever (11, 37.9%). Clinically, 20 patients (39.2%) did not present with hyperbilirubinemia or increased creatinine levels. Two patients (3.9%) died, both from respiratory failure. Fifteen patients (29.4%) clinically diagnosed with leptospirosis at sites were negative based on IgM ELISA and/or PCR at the Reference Laboratory.

**Conclusions:**

Leptospirosis remains an important cause of hospitalization in Indonesia. It can have diverse clinical presentations, making it difficult to differentiate from other common tropical infections. PCR combined with ELISA is a powerful alternative to the cumbersome gold-standard microscopic agglutination test, particularly in resource-limited settings.

## Background

Leptospirosis is a significant, but often overlooked, bacterial infection endemic in tropical and sub-tropical regions. Though its detected burden is low relative to other tropical diseases in Indonesia, most notably dengue fever, leptospirosis remains a significant public health problem, especially in regions that experience heavy rain and flooding [1]. This potentially life-threatening but treatable zoonosis caused an estimated 895 human cases in Indonesia during 2018, with a case fatality rate of 17.8%, according to official Ministry of Health (MoH) reports [2] However, this case number is certainly a severe underestimate of leptospirosis in Indonesia given that the annual morbidity of leptospirosis in the population was recently estimated at 39.2 per 100,000 [3]. The significant discrepancy between observed cases and case estimates highlights the poor understanding of the leptospirosis burden in Indonesia and the clear need for additional epidemiological data.

Physicians in Indonesia are generally unfamiliar with the clinical presentations of leptospirosis, which include non-specific anicteric and flu-like manifestations in the majority of cases. Associated manifestations such as acute fever, headache, chills, and myalgia overlap with those of dengue and typhoid fevers [4], which are more common than leptospirosis in Indonesia. Timely diagnosis of this treatable bacterial infection is critical for appropriate case management. Untreated cases are at an increased risk of progression to the severe manifestation of Weil’s disease, which has a > 70% case fatality rate [3].

In addition to diverse clinical symptoms and overlapping presentation with other endemic infections, poor access to accurate diagnostic tests complicates the diagnosis of leptospirosis [5, 6]. The serological ‘gold-standard’ microscopic agglutination test (MAT), which requires considerable resources and trained staff [5, 6], is only available at three centers in all of Indonesia. Alternative tests such as qPCR, which can detect infection early in the course of the disease, are seldom available in resource-limited settings where leptospirosis is common. The resulting under-diagnosis of leptospirosis perpetuates low awareness and poor understanding of disease epidemiology [6].

To characterize the clinical manifestations, diagnostic challenges, and outcomes of endemic leptospirosis requiring hospitalization in Indonesia, we analyzed demographic, clinical, and laboratory data from patients enrolled in a multi-site observational study conducted at eight tertiary care hospitals across the country from 2013 to 2016.

## Methods

### Study population

Patients were enrolled in the Etiology of Acute Febrile Illness Requiring Hospitalization (AFIRE) cohort study, conducted by the INA-RESPOND (Indonesia Research Partnership on Infectious Diseases) research network [7] in Indonesia from 2013 to 2016. The AFIRE study recruited patients at eight tertiary-care hospitals who presented for evaluation of acute fever, were at least 1 year-old, were hospitalized within the past 24 h, and had not been hospitalized within the past 3 months. Clinical information and biological specimens were collected at enrollment, 14–28 days after enrollment, and 3 months after enrollment. Details of the AFIRE study have been previously described [8].

### Reference laboratory evaluation for leptospirosis

Serological assays for anti-*Leptospira* IgM were performed on both acute and convalescent samples in parallel. As the sensitivities of IgM-specific ELISAs range from 43 to 90.8% [9, 10], ELISA IgM kits from two manufacturers were used. The SERION ELISA *classic* Kit (Cat# ESR125G, Institut Virion/Serion GMBH-Germany) was performed according to the manufacturer’s instructions. Antibody activity was automatically calculated using SERION software. For IgM, < 15 U/mL was interpreted as negative, 15–20 U/mL as borderline, and > 20 U/mL as positive. Additionally, the PanBio *Leptospira* IgM ELISA (PanBio Cat# 02PE10, Standard Diagnostics Inc., Gyeonggi-do, Korea) was performed following the manufacturer’s instructions. An index value was calculated by dividing the sample absorbance by the cut-off value. An index value of < 0.9 was interpreted as negative, index > 0.9 to < 1.1 as borderline, and index > 1.1 as positive.

Acute specimens of patients sero-positive for *Leptospira* were further tested using an in-house TaqMan real-time PCR assay to confirm the presence of pathogenic *Leptospira* DNA. Briefly, DNA was extracted from 200 μl of buffy coat or plasma from acute specimens using the QIAamp DNA Blood Mini Kit (Qiagen, de Hilden Germany). Extracts were then used to perform a TaqMan real time PCR assay targeting the *rrs* and *lipL32* genes of *Leptospira spp.* Amplifications were done using TaqMan Fast Universal PCR Master Mix (Thermo Fisher Scientific) and run on an Applied Biosystem 7500 Fast real-time PCR instrument. Primers, probes, and procedures are described in detail in the references (11, 12). When results differed between the *rrs* and *lipL32* qPCR assays, positive results from each qPCR assay were considered valid when supported by serological evidence. The reference standard MAT was not performed due to study limitations. Although one hospital in Indonesia has the capability of conducting MAT, it was not utilized in the AFIRE study.

### Case definition of leptospirosis

Taking into consideration the diverse clinical manifestations of leptospirosis, the limited availability of diagnostics, and the need for early case detection and treatment, the U.S. Centers for Disease Control and Prevention developed a leptospirosis case definition [13]. For this study, the case definition was adapted as follows:

*Probable Leptospirosis* is a clinically suspected leptospirosis case with a high titer (≥1600) of ELISA IgM antibodies by both Serion and PanBio assays; *Confirmed Leptospirosis* is a clinically suspected leptospirosis case with a positive qPCR result from plasma or buffy coat and/or a four-fold increase or sero-conversion in the convalescent specimen of ELISA IgM antibodies by Serion and/or PanBio assays.

### Statistical analysis

Data were collected in OpenClinica v.3.1 (OpenClinica, LLC) and analyzed using STATA v.15.1 (StataCorp LLC). Proportions were compared between groups using the chi-square test. The t-test was used to compare means between groups.

## Results

### Epidemiology

**Fig. 1:**
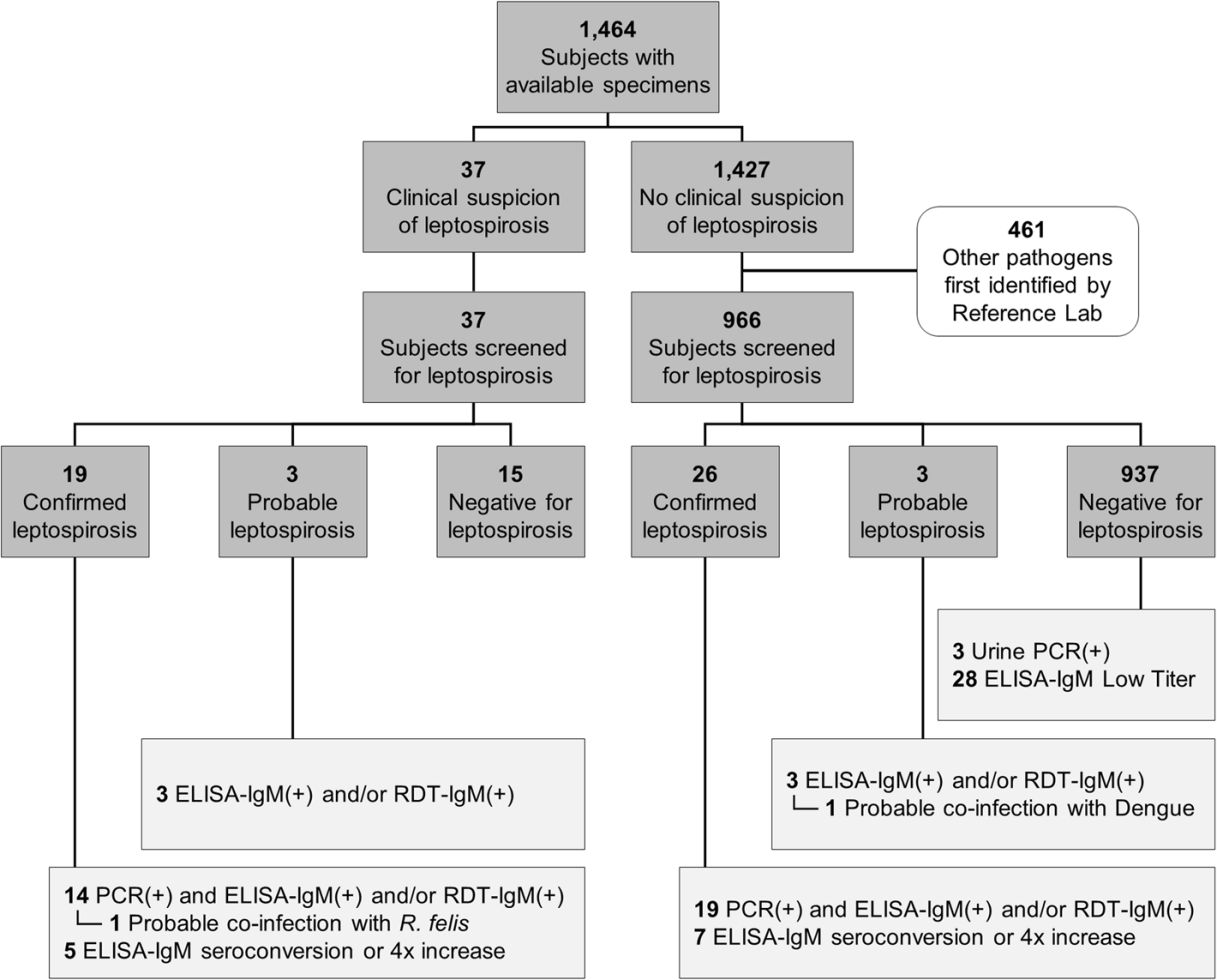
Schema for identifying leptospiral infections amongst patients hospitalized with acute fevers

The AFIRE study enrolled 1486 patients. Of 1464 with available specimens, 37 were clinically suspected of having leptospirosis. Of the 1427 not suspected of having leptospirosis, 966 had no pathogen identified. Samples from patients suspected of having leptospirosis and from patients with no pathogen identified were tested for leptospirosis as described above. Evidence of *Leptospira* infection was found in 22 of the 37 clinically suspected patients and 29 of the 966 without an identified pathogen (Fig. 1).

**Fig. 2:**
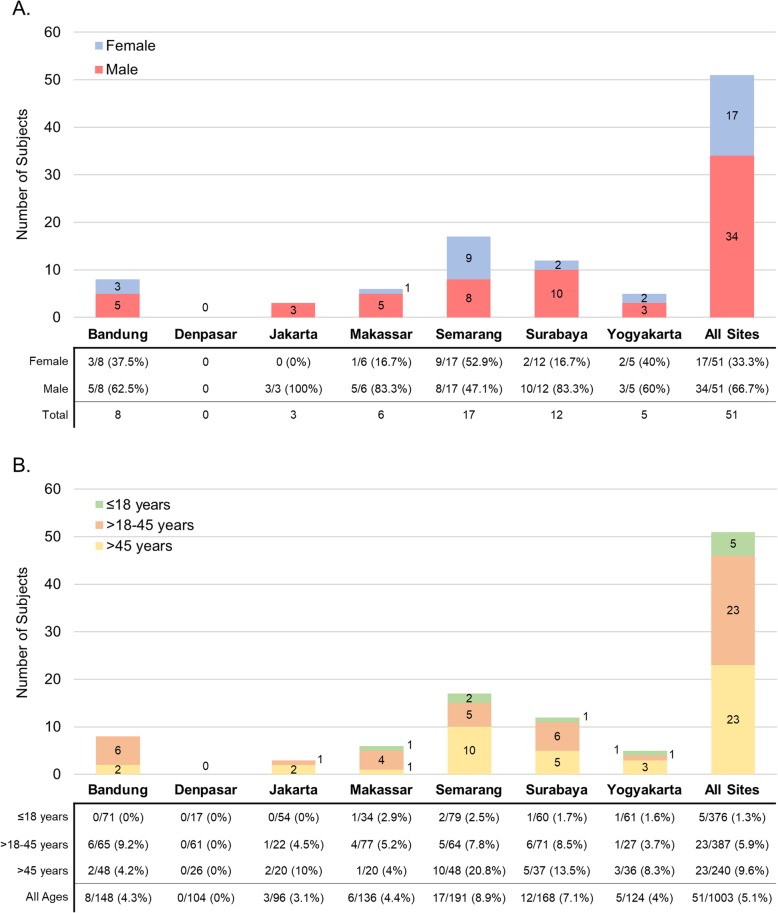
Distribution of leptospirosis cases in each city by gender and age categories

The 51 leptospirosis cases constitute 5.1% of tested patients and 3.5% of enrolled patients. Most cases were from Semarang (17/51, 33.3%) and Surabaya (12/51, 23.5%), which is not a statistically significant distribution compared to the other sites (1.9, *p* = 0.17). Additionally, the proportion of leptospirosis amongst tested patients was significantly higher in Semarang (17/191, 8.9%) and Surabaya (12/168, 7.1%) compared to other sites (14.191, *p* = 0.028). There were no cases from Denpasar. Leptospirosis tended to be more common in men (34/51, 67%) (3.722, *p* = 0.054) and in adults > 45 years (9.6%) compared to adults > 18–45 years (5.9%) (2.939, *p* = 0.086) and pediatrics 1- ≤ 18 years (1.3%.) (23.208, *p* < 0.001). The distribution of cases in each city grouped by gender and age categories is shown in Fig. 2.

### Laboratory diagnostic tests

Thirty-three of the 51 leptospirosis cases were confirmed by PCR. *Leptospira* DNA was detected in blood and urine for one patient, blood-only for 29 patients, and urine-only for three patients. In these three cases, *Leptospira* ELISA IgM results were supportive. Twelve cases were confirmed by *Leptospira* ELISA IgM sero-conversion or four-fold increase. Six probable cases showed high IgM titer (1/1600) in the acute specimen by ELISA. The onset of illness in PCR-positive patients was significantly shorter than those who were PCR-negative (mean (SD): 4.58 (1.66) days vs. 5.78 (2.44) days) (t(49) = (− 2.088); *p* = 0.042). One patient with detected *Leptospira* DNA and sero-conversion of both *Leptospira* IgM antibodies was also positive for *Rickettsia felis*, suggesting dual infection.

Three patients diagnosed with urinary tract infections (UTI) had detectable *Leptospira* DNA in the urine. As diagnosis of leptospirosis was not supported by *Leptospira* IgM or *Leptospira* DNA in the blood, the 3 patients were not included as acute cases. The 28 patients with non-increasing detectable ELISA IgM antibodies in lower titers (1:100–1:400) were also not included as cases.

### Clinical presentations

At the hospital sites, leptospirosis was not diagnosed in 29 (56.9%) of the 51 cases detected by the Reference Laboratory (missed cases). Yogyakarta, Makassar, and Surabaya had the highest missed case rates (100, 83.3, and 83.3%, respectively). These cases were initially diagnosed as dengue fever (11/29, 37.9%), typhoid fever (4/29, 13.8%), UTI (5/29, 17.2%), community-acquired pneumonia (3/29, 10.3%), diarrhea (2/29, 6.9%), sepsis, undifferentiated fever, pharyngitis, and ulcer (1/29, 3.4%, each). The characteristics of Reference Laboratory-confirmed leptospirosis cases according to initial clinical diagnoses at sites are shown in Table 1.

**Table 1:** Characteristics of confirmed leptospirosis cases according to initial clinical diagnoses at sites

	Clinical Diagnosis at Site						
	Dengue (*n* = 11)	Typhoid fever (*n* = 4)	UTI (*n* = 5)	CAP (*n* = 3)	Other^a^ (*n* = 6)	Leptospirosis (*n* = 22)	Total (*n* = 51)
Demography							
Gender (male: female)	10:1	1:3	2:3	2:1	3:3	16:6	34:17
Adult: Pediatric^b^	9:2	4:0	4:1	3:0	4:2	22:0	46:5
Age, median (range)	29 (5.3–58.1)	42.3 (19.2–60.4)	27 (10–61.4)	62.5 (59.7–64.4)	47 (13–85)	41.7 (20.8–62.1)	41.2 (5.3–85)
Signs & Symptoms, *N* (%)							
Anorexia	3 (27.3)	2 (50)	0	1 (33.3)	3 (50)	7 (31.8)	16 (31.4)
Chills	4 (36.4)	2 (50)	3 (60)	2 (66.7)	1 (16.7)	9 (40.9)	21 (41.2)
Lethargy	2 (18.2)	1 (25)	1 (20)	0	1 (16.7)	10 (45.5)	15 (29.4)
Icterus	2 (18.2)	0	0	0	1 (16.7)	6 (27.3)	9 (17.6)
Decrease of consciousness	0	0	0	0	1 (16.7)	0	1 (1.9)
Headache	5 (45.5)	3 (75)	3 (60)	1 (33.3)	3 (50)	13 (59.1)	28 (54.9)
Dizziness	2 (18.2)	1 (25)	3 (60)	2 (66.7)	2 (33.3)	6 (27.3)	16 (31.4)
Cough	2 (18.2)	0	1 (20)	1 (33.3)	2 (33.3)	9 (40.9)	15 (29.4)
Shortness of breath	0	0	0	0	0	4 (18.2)	4 (7.8)
Epigastric pain	0	2 (50)	2 (40)	1 (33.3)	1 (16.7)	8 (36.4)	14 (27.5)
Abdominal pain	2 (18.2)	0	0	0	1 (16.7)	5 (22.7)	8 (15.7)
Nausea	8 (72.7)	4 (100)	4 (80)	3	4 (66.7)	17 (77.3)	40 (78.4)
Vomiting	7 (63.6)	3 (75)	3 (60)	2 (66.7)	5 (83.3)	12 (54.5)	32 (62.7)
Constipation	2 (18.2)	2 (50)	0	0	1 (16.7)	0	5 (9.8)
Diarrhea	5 (45.5)	0	2 (40)	2 (66.7)	3 (50)	9 (40.9)	21 (41.2)
Arthralgia	2 (18.2)	0	4 (80)	2 (66.7)	0	12 (54.5)	20 (39.2)
Myalgia	1 (9.1)	1 (25)	3 (60)	1 (33.3)	1 (16.7)	15 (68.2)	22 (43.1)
Skin rash	0	0	0	1 (33.3)	0	1 (4.5)	2 (3.9)
*Conjunctival suffusion*	*2 (18.2)*	*0*	*0*	*0*	*1 (16.7)*	*11 (50)*	*14 (27.5)*
*Rhonchi*	*0*	*0*	*0*	*2 (66.7)*	*0*	*4 (18.2)*	*6 (11.8)*
*Pruritus*	*0*	*1 (25)*	*0*	*0*	*0*	*0*	*1 (1.9)*
*Gastrocnemius pain*	*1 (9.1)*	*0*	*0*	*0*	*1 (16.7)*	*6 (27.3)*	*8 (15.7)*
*Oliguria*	*1 (9.1)*	*0*	*0*	*0*	*0*	*3 (13.6)*	*4 (7.8)*
*Brown-coloured urine*	*0*	*0*	*0*	*0*	*0*	*7 (31.8)*	*7 (13.7)*
Haematology & Chemistry Results, N (%) / N/tested (%)							
Leukopenia	0	0	1 (20)	0	1 (16.7)	0	2 (3.9)
Normal leucocyte count	10 (90.9)	3 (75)	2 (40)	2 (66.7)	0	6 (27.3)	23 (45.1)
Leukocytosis	1 (9.1)	1 (25)	2 (40)	1 (33.3)	5 (83.3)	16 (72.7)	26 (50.9)
Platelet ≤150,000/mm^3^	8 (72.7)	2 (50)	1 (20)	2 (66.7)	1 (16.7)	16 (72.7)	30 (58.8)
Granulocytosis > 80%	8/10 (80)	2/3 (66.7)	1/4 (25)	2/2 (100)	3/5 (60)	10/12 (83.3)	26/36 (72.2)
Lymphopenia < 20%	9/10 (90)	4/4 (100)	2/4 (50)	2/2 (100)	5/5 (100)	13/13 (100)	35/38 (92.1)
Total bilirubin > 1 mg/dL	3/11 (27.3)	1/3 (33.3)	0/4	1/3 (33.3)	0/5	15/22 (68.2)	20/48 (41.7)
Bilirubin direct > 0.65 mg/dL	2/11 (18.2)	1/3 (33.3)	0/4	0/3	0/5	16/22 (72.7)	19/48 (39.6)
AST^c^ > 100	1/11 (9.1)	0/3	0/4	0/3	0/6	7/20 (35)	8/47 (17)
ALT^d^ > 100	0/11	0/3	0/4	0/3	0/6	3/22 (13.6)	3/47 (6.4)
Creatinine > 1.4 mg/dL	3/11 (27.3)	0/3	2/4 (50)	2/3 (66.7)	0/5	15/22 (68.2)	22/48 (45.8)
Diagnostic tests at sites, N/ tested (%)							
Dengue IgM positive	2/6 (33.3)	2/4 (50)	0/1	0/1	0/1	0/5	4/18 (22.2)
Tubex TF®^e^	2/5 (40)	4/4 (100)	1/2 (50)	0/1	1/3 (33.3)	2/5 (40)	10/20 (50)
Leptospira IgM positive	0/1	0/4	0/1	0/1	0/1	18/22 (81.8)	18/25 (72)
Reference Laboratory tests, N (%)							
Dengue & S. typhi	1 (9.1)	0	0	0	0	0	1 (1.9)
Leptospira PCR & Serology	6 (54.5)	2 (50)	2 (40)	3 (100)	3 (50)	12 (54.5)	28 (54.9)
Leptospira PCR only	2 (18.2)	0	0	0	1 (16.7)	2 (9.1)	5 (9.8)
Leptospira serology only	3 (27.3)	2 (50)	3 (60)	0	2 (33.3)	8 (36.4)	18 (35.3)
Outcome, N (%)							
Death	1 (9.1)	1 (25)	0	0	0	0	2 (4)

^a^diarrhea (2), sepsis (1), pharyngitis (1), ulcus pedis (1), undifferentiated fever (1)

^b^adult aged ≥18 year old; pediatric aged ≥1 - < 18 year old

Signs and symptoms in italics were not obtained from all patients as they were not included in the case report form. They were extracted from chart review

^c^AST: aspartate aminotransferase

^d^ALT: alanine aminotransferase

^e^TUBEX TF® is a rapid diagnostic test to detect IgM antibodies against *Salmonella typhi*. Scores of 4–6 were considered positive

Leptospirosis could not be confirmed in 15 of 37 (40.5%) subjects clinically diagnosed at the sites, mostly from Semarang 12/15 (80%). Reference Laboratory testing revealed evidence of *Rickettsia typhi* in four cases and *Staphylococcus aureus*, chikungunya virus, and dengue virus in one case each. Etiologies remained unknown in eight cases. All 15 patients were adults, with a median age of 36.8 years (range 20.2–62.8), and were predominantly male (10:5). Hematological findings consistent with leptospirosis, such as leukocytosis, thrombocytopenia, and granulocytosis, were found in 46.7% (7/15), 53.3% (8/15), and 42.9% (6/14) of the cases, respectively. Hyperbilirubinemia and increased creatinine levels were reported in 33.3% (5/15) and 20% (3/15) of the cases, respectively. In 11 of the cases, leptospirosis could not be confirmed by further testing despite observing low ELISA IgM antibody titers for one case and positive rapid IgM tests for ten cases. In 3 cases, rapid IgM was negative, and in one case, rapid IgM was not done. In these 4 cases, the diagnosis of leptospirosis was based solely on clinical judgement. Single MAT was done in 5 of 15 patients as part of study-independent standard-of-care testing, and none were positive. The INA-RESPOND Reference Laboratory found ELISA IgM antibody and qPCR assays to be negative in 12 patients. Low IgM titers were detected in three patients, but none were further supported by increasing IgM titers or *Leptospira* nucleic acid detection. Details of the clinical presentations and diagnostic assays from each patient are shown in Table 2.

**Table 2:** Characteristics of cases clinically misdiagnosed as leptospirosis

No	*Leptospira* symptoms	Other symptoms^a^	*Leptospira* Standard of Care Results at sites	*Leptospira* Reference Lab	Final pathogen identified & Lab results	
1	Icterus	2,5,6,9,13,14,17,18, 19,20,21, 22	Rapid IgM (neg)	PCR negative IgM ELISA negative	UNK	
2	Icterus, suffusion, brown-colored urine	2,4,5,6,7,10,13,14,16,17,18,19,20	IgM ELISA, low titer (Conv)	PCR negative IgM ELISA negative	*R. typhi*	PCR Positive IgM (0.9–4.7) IgG (2.8–15.2) IFA Pos
3		1,5,10,12	Not done	PCR negative IgM ELISA negative	UNK	
4	Gastrocnemius pain, Icterus	2,5,10,13,16,19,20, 22	Rapid IgM negative	PCR negative IgM ELISA negative	UNK	
5	Flank pain	2,13,14	Rapid IgM positive, MAT negative	PCR negative IgM ELISA negative	*R. typhi*	PCR Negative IgM (1.3–12.8) IgG (0.4–3.2) IFA Pos
6		2,5,6,9,13,19	Rapid IgM positive	PCR negative IgM ELISA negative	UNK	
7		3,4,6,12,13,14,17,18,20	Rapid IgM positive	PCR negative IgM ELISA negative	*R. typhi*	PCR Positive IgM (2.3–8.9) IgG (1.5–10.1) IFA Pos
8	Icteric, suffusion	1,4,11,13,19	Rapid IgM positive, MAT negative	PCR negative IgM ELISA negative	*S. aureus*	Blood culture
9		2,3,4,13,16,17,19,20,22	Rapid IgM negative	PCR negative IgM ELISA titer 1:100 convalescent sera	Chikungunya	PCR Negative IgM (6.4–3.3) IgG (0.2–3.0)
10	Suffusion	3,5,8,10,13.14.20	Rapid IgM positive	PCR negative IgM ELISA negative	UNK	
11	Gastrocnemius pain, brown-colored urine, suffusion	2,5,9, 16,17,18,19	Rapid IgM positive, MAT negative	PCR negative IgM ELISA borderline	UNK	
12	Brown-colored urine, suffusion	2,3,4,5,10,13,14,18,20	Rapid IgM positive, MAT negative	PCR negative IgM ELISA negative	*R. typhi*	PCR Negative IgM (0.4–1) IgG (2.7–13.4) IFA Pos
13		6,13,20	Rapid IgM positive	PCR negative IgM ELISA borderline	Dengue	PCR Positive DENV-3 NS1 positive IgM (6.335–3.819) IgG (12.371–12.931)
14	Suffusion	3,16,23,19,20	Rapid IgM positive, MAT inconclusive	PCR negative IgM ELISA negative	UNK	
15	Gastrocnemius pain	13,14,15	Rapid IgM positive	PCR negative IgM ELISA titer 1:100 in acute and convalescent sera	UNK	

^a^1: decrease of consciousness; 2: chills; 3: lethargy; 4: anorexia; 5: headache; 6: dizziness; 7: epistaxis; 8: runny nose; 9: sore throat; 10: cough; 11: rhonchi; 12: shortness of breath; 13: nausea; 14: vomiting; 15: diarrhea; 16: constipation; 17: abdominal pain; 18: epigastric pain; 19: arthralgia; 20: myalgia; 21: itchy; 22: ecchymosis; 23: hematemesis

The median onset of fever was 5 days (range 2–11) prior to presentation. In the 49 of 51 Reference Laboratory-diagnosed patients who survived, the median hospitalization time was 6 days (range 3–18). All 51 cases received antibiotics, including ceftriaxone (26 cases), ciprofloxacin (10 cases), doxycycline (5 cases), ampicillin, azithromycin, levofloxacin, cefotaxime, and cefixime (1 case each), a combination of antibiotics (4 cases), and unknown antibiotics (1 case). Leptospirosis patients correctly diagnosed at sites were hospitalized longer than those misdiagnosed (mean (SD): 8.91 (3.78) days vs. 5.28 (1.73) days, t(49) = 4.593; p = < 0.0001).

The two leptospirosis patients (4%) who died during hospitalization were misdiagnosed cases. The first was a male clinically diagnosed with dengue hemorrhagic fever, and the second was a diabetic female clinically diagnosed with typhoid fever. Both came to the hospital 3 days after fever onset and died 2 days later. Details of their clinical and laboratory findings are shown in Table 3.

**Table 3:** Characteristics of the two fatal leptospirosis cases that were misdiagnosed

	Patient 1 (Surabaya)	Patient 2 (Makassar)
Gender, age range (years)	Male, 45–50	Female, 60–65
Comorbidities	No comorbidities	Diabetes mellitus
Signs/Symptoms	Fever (3 days), nausea, vomiting, diarrhea	Fever (3 days), chills, headache, dizzy, nausea, vomiting
Hematology profiles		
Hb (mg/dL)	11.5	13
Hematocrit (%)	32.9	38.7
Leukocyte count (/mm^3^)	9600	9500
Granulocytes (%)	92%	Not available
Lymphocytes (%)	3.8%	6.9%
Platelet (/mm^3^)	45,000	118,000
Chemistries		
Bilirubin total, direct, indirect (mg/dL)	0.9; 0.6; 0.3	
AST^a^ (IU/mL)	139	93
ALT^b^ (IU/mL)	33	64
Creatinine (mg/dL)	4.1	1.1
Diagnostic tests at the hospital		
Dengue test	Not tested	Negative IgM/IgG
*Salmonella typhi*	Not tested	Tubex TF® test positive [6]
Diagnosis at the hospital	Dengue hemorrhagic fever grade I	Typhoid fever
Antibiotics	Ceftriaxone	Cefotaxime, intra-venous
Cause of death, hospitalization day	Respiratory failure, 2 days	Respiratory failure, 2 days
Diagnostic test at the reference laboratory (only acute specimen available)		
Dengue virus	RT-PCR, NS1, IgM negative, IgG positive	RT-PCR, NS1, IgM negative, IgG positive
*Salmonella typhi*	Blood culture, PCR, ELISA IgM, IgG negative	Blood culture, PCR, ELISA IgM and IgG negative
*Leptospira spp*	PCR positive (ct value = 30)	PCR positive (ct value = 19)
	ELISA IgM, IgG negative	ELISA IgM, IgG negative

^a^*AST* aspartate aminotransferase

^b^*ALT* alanine aminotransferase

## Discussion

Acute infection with *Leptospira* was identified in 3.5% of our cohort of patients hospitalized with acute febrile illness in Indonesia. This number could be an under-reporting of the total number of cases in the cohort given that febrile cases confirmed as dengue virus infections were not examined for co-infection with *Leptospira* due to study resource limitations. We know of no other recent studies reporting the incidence of leptospirosis from seven large cities across Indonesia. Previously published reports have been from Jakarta in 1993–1995 [14], Papua in 1997–2000 [14], Semarang in 1995–1996 and 2005–2009 [15–17], Tangerang in 2015 [18], and returning travelers from Sumatra and Bali in 2008 and 2013 [19, 20]. Rates of acute leptospirosis in our study ranged from 0% in Denpasar to 8.9% in Semarang, likely reflecting country-wide variations in endemicity. However, prior reports of leptospirosis in travelers returning from Bali suggest that our study results may not be representative or that the epidemiology of leptospirosis is changing. Our findings are consistent with MoH data in which only 8 of 34 provinces in Indonesia (6 on Java island) reported 895 leptospirosis cases in 2018, with the highest incidence being in Central (613 cases) and East (128 cases) Java provinces [2] where our 3 sites in Semarang (17/51 cases), Yogyakarta (5/51 cases), and Surabaya (12/51 cases) are located. As we found leptospirosis in Makassar, where there are no documented cases according to the MoH report [2], we recommend continued surveillance for leptospirosis, particularly in provinces where its epidemiology has not been characterized. This will facilitate the development of targeted risk reduction strategies. Missed cases in our study were predominantly mild and without pathognomonic signs or symptoms of leptospirosis. There was a surprisingly high number of patients with GI symptoms such as nausea (40/51, 78.4%) and vomiting (32/51, 62.7%), including in both fatal cases. These symptoms were also frequently found in our dengue, typhoid fever and rickettsioses cases (unpublished data). Observing these unexpected GI symptoms with leptospirosis should raise the index of suspicion among clinicians seeing acute febrile patients. As reports on the frequency of GI symptoms in leptospirosis vary [4, 21–23], further study is needed to better characterize the clinical manifestations of leptospirosis in endemic areas.

Missed cases tended to have shorter hospitalizations than accurately diagnosed cases. The frequency of missed cases we observed highlights the difficulty in diagnosing mild leptospirosis by its non-specific and variable clinical presentation. However, retrospective analyses revealed the presence of several typical markers, such as mild increases in bilirubin, transaminases, creatinine, and granulocytes, at enrollment in some patients. Thus, thorough laboratory and clinical evaluations remain key components of the diagnostic approach, particularly when other infections have been ruled out. Doxycycline is the recommended treatment for leptospirosis, and empiric administration against suspect or probable leptospirosis cases may prevent progression of the disease. Fortunately, doxycycline is indicated for both leptospirosis and rickettsiosis, so the drug would cover both frequently co-circulating diseases if a specific diagnosis was uncertain [24]. Intravenous penicillin should be initiated for clinically severe forms of the disease, which may contribute to decreasing mortality. In most cases, ceftriaxone may be used as an alternative [25, 26].

Similar diagnostic challenges have been reported in other nearby countries, including Thailand, where most missed cases were initially diagnosed as dengue fever, other viral infections, or scrub typhus [27]. In Semarang, where leptospirosis has been more frequently reported [15, 16], the disease was over-diagnosed. Over-diagnosis is common when leptospirosis is prevalent [28], particularly when patients present with typical features of leptospirosis such as conjunctival suffusion or icterus. In 4 of 15 clinically-presumed cases, rickettsioses were confirmed, suggesting that these features are not exclusive to leptospirosis. Other diseases that may present with jaundice or acute kidney injury include cholecystitis, hepatitis, arbovirus infection, malaria, hantavirus infection, and rickettsioses [29, 30]. As such, laboratory testing is needed to help distinguish the etiology.

Though a causal pathogen could not be confirmed in eight cases, it is unlikely that we missed leptospirosis given the extensive testing conducted. qPCR using primers for *rrs* and *lipL32* genes was performed on both buffy coat and plasma [11, 12, 31], and two ELISAs for IgM, both with excellent sensitivity and specificity compared to MAT [10, 32], were performed using acute and convalescent specimens. However, the lack of diagnostic specificity with some tests, especially rapid tests [10], may contribute to the over-diagnosis of leptospirosis. Furthermore, IgM antibodies from past infections are frequently detected in people living in endemic areas, which complicates assay interpretation, particularly when a single specimen is used [33]. Our classification of 3 patients with positive urine PCR but low/undetectable IgM antibody titers as not having acute leptospirosis is consistent with prior findings that *Leptospira* bacteria may persist in urine for several months after recovery [34].

Our findings are consistent with previous reports that leptospirosis is less frequent in women and children. The findings are also consistent with reports of missed childhood cases, as all childhood cases in our cohort were missed by clinicians presumably due to non-specific clinical presentations [27, 35, 36]. It is unclear why leptospirosis is more severe in adults than children. Contributing factors may include host susceptibility [37], *Leptospira* virulence [12], and changes in the immune response [38]. The observed lower frequency of leptospirosis in women is thought to be related to lower levels of outdoor activities compared with men [39], lower levels of leptospiremia [40], and clinically less severe disease [41]. However, in our study the proportion of women correctly diagnosed with leptospirosis and missed by sites was similar, suggesting the lower rates in women are not related to disease severity.

We also found evidence of co-infections with rickettsioses, as confirmed by molecular and IFA assays, and leptospirosis, as confirmed by qPCR and sero-conversion of IgM antibodies. Co-infection with these two pathogens has been reported previously and is plausible given that both share rodent vectors [42].

Reliance on MAT, the current gold-standard for serology, is impractical in certain settings due to technical requirements and expense. Since an accurate rapid diagnostic test for leptospirosis is not available, molecular tests and ELISAs for IgM can be used to inform medical management in resource-limited settings, as has also been suggested previously [43] .

Since this study was part of a larger study to identify the etiologies of acute fever requiring hospitalization, a thorough assessment of leptospirosis-associated features was not specifically conducted. This limitation means that signs typically considered indicative of *Leptospira* infection, such as suffusion and gastrocnemius pain, may have been under-assessed. Furthermore, co-infection with leptospirosis was not assessed in patients whose current febrile illness was already attributed to another pathogen by the Reference Laboratory. This may have resulted in an under-reporting of the true leptospirosis burden in this cohort. Another limitation is that we did not perform MAT, the gold standard for serological evaluation of *Leptospira* infection. It remains unlikely that we missed leptospirosis cases for the several reasons described above, but we lack information about the infecting serogroups, which could inform public health strategies and management of animal reservoirs. Serogroup determination would be helpful for assessment of the relationship between clinical course, epidemiology, and serogroups in Indonesia, which has not yet been well-characterized. Lastly, this study was conducted only in large Indonesian cities instead of throughout the archipelago, limiting the generalizability of our findings to the entire population.

## Conclusions

Leptospirosis is an important cause of fever leading to hospitalization in Indonesia. The high proportion of leptospirosis cases missed at several sites highlights the needs for increased clinician awareness of possible clinical presentations and appropriate diagnostic approaches. There is an imminent need for the development of accurate rapid diagnostics for leptospirosis and other co-endemic pathogens. In the absence of reliable rapid diagnostic tests, leptospirosis should be included by clinicians as an important differential diagnosis of acute febrile illnesses. Dissemination of clinical information and research findings, delineation of optimal empiric management, surveillance activities to inform risk reduction, and improved access to reliable point-of-care diagnostics for leptospirosis should be prioritized by policymakers.

## Data Availability

The data supporting the conclusions of this article are available upon request. Please contact dr. Herman Kosasih (email: hkosasih@ina-respond.net).

## Abbreviations

AFIRE: The Etiology of Acute Febrile Illness Requiring Hospitalization
DNA: Deoxyribonucleic acid
ELISA: Enzyme-linked immunosorbent assay
INA-RESPOND: Indonesia Research Partnership on Infectious Diseases
MAT: Microscopic agglutination test
MoH: Ministry of Health
qPCR: Quantitative real-time polymerase chain reaction
UTI: Urinary Tract Infection
WHO: World Health Organization
